# An Uncommon Association of Familial Partial Lipodystrophy, Dilated Cardiomyopathy, and Conduction System Disease

**DOI:** 10.1177/2324709616658495

**Published:** 2016-07-15

**Authors:** Ragesh Panikkath, Deepa Panikkath, S. Sanchez-Iglesias, D Araujo-Vilar, Joaquin Lado-Abeal

**Affiliations:** 1 Texas Tech University Health Sciences Center, Lubbock, TX, USA; 2 Complexo Hospitalario de Santiago de Compostela, University of Santiago, Santiago, Spain

**Keywords:** familial partial lipodystrophy, lipodystrophy, cardiomyopathy, conduction disorders

## Abstract

A 46-year-old African American woman presented with severe respiratory distress requiring intubation and was diagnosed with nonischemic cardiomyopathy. She had the typical phenotype of familial partial lipodystrophy 2 (FPLD2). Sequence analysis of *LMNA* gene showed a heterozygous missense mutation at exon 8 (c.1444C>T) causing amino acid change, p.R482W. She later developed severe coronary artery disease requiring multiple percutaneous coronary interventions and coronary artery bypass surgery. She was later diagnosed with diabetes, primary hyperparathyroidism, and euthyroid multinodular goiter. She had sinus nodal and atrioventricular nodal disease and had an implantable cardioverter defibrillator implantation due to persistent left ventricular dysfunction. The device eroded through the skin few months after implantation and needed a re-implant on the contralateral side. She had atrial flutter requiring ablation. This patient with FPLD2 had most of the reported cardiac complications of FPLD2. This case is presented to improve the awareness of the presentation of this disease among cardiologists and internists.

## Introduction

Dunnigan-type familial partial lipodystrophy 2 (FPLD2; MIM: #151660) is a genetic disease caused by mutations in lamin A/C (*LMNA*) gene (ENSG00000160789). The phenotype is characterized by atrophy of subcutaneous adipose tissue on extremities and trunk with fat deposition in the face and neck that occasionally gives cushingoid appearance to patients.^1^ Patients with FLPD2 often present insulin resistance and related metabolic complications such as diabetes and dyslipidemia.^1^ However nonischemic cardiomyopathy and heart conduction defects are rarely the first clinically relevant manifestation in these patients.^2^ This is a case report of a patient with Dunnigan disease that was confirmed by mutational analysis of *LMNA* gene. The patient presented with a cardiac conduction system disease and nonischemic cardiomyopathy initially but later developed severe coronary artery disease requiring multiple stents and coronary artery bypass surgery.

## Case Description

**Figure 1.**
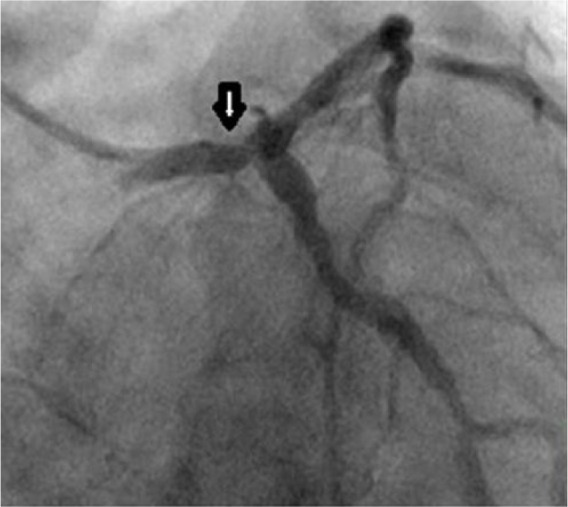
Coronary angiography showing significant disease in the distal left main coronary artery.

A 46-year-old African American woman presented to the emergency room with respiratory distress requiring intubation. She was found to have severe pulmonary edema on initial evaluation. A transthoracic echocardiogram showed global hypokinesis of the left ventricle and severe systolic dysfunction, with an ejection fraction of 30%. A myocardial perfusion test showed no evidence of stress-induced ischemia, and she was diagnosed with nonischemic cardiomyopathy. Her physical appearance was remarkable for prominent loss of subcutaneous adipose tissue in the extremities and trunk but excessive adipose tissue deposition in her neck with severe acanthosis nigricans suggestive of partial lipodystrophy. Sequence analysis of *LMNA* gene showed a heterozygous missense mutation at exon 8 (c.1444C>T) causing amino acid change, p.R482W. Her glycosylated hemoglobin was 5.8, indicating that she had prediabetes. One year later a coronary angiogram performed due to retrosternal chest pain showed mild coronary artery disease in left anterior descending artery (LAD) and right coronary artery with an ejection fraction of 25%. The patient was continued on medical therapy. At this point she was diagnosed with diabetes mellitus (glycosylated hemoglobin 6.6%). A lipid profile showed total cholesterol 215 mg/dL, triglycerides 260 mg/dL, low-density lipoprotein 124 mg/dL, high-density lipoprotein 39 mg/dL, and very low density lipoprotein 52 mg/dL. Primary hyperparathyroidism and euthyroid multinodular goiter were also diagnosed. She continued having multiple recurrences of chest pain. A nuclear perfusion scan showed inferior wall scar with moderate amount of peri-infarct ischemia. A series of coronary angiograms performed during a period of 12 months showed significant coronary artery disease: firstly of right coronary artery (90%) that required stenting followed by LAD (80%) that was stented, distal left main coronary (Figure 1), proximal left anterior descending and obtuse marginal (OM) managed by coronary artery bypass graft (CABG) surgery. An angiogram performed for recurrence of chest pain 4 months after the CABG showed inadequate flow from the left internal mammary artery graft to the LAD but patent saphenous vein grafts to the OM and posterior descending artery. Stenting was done across the left main to the LAD. She improved clinically following this procedure.

Two years after her initial presentation, she had asymptomatic transient complete heart block, which was thought to be related to β-blockers. This was managed conservatively. Four years after the initial presentation, she had symptomatic sinus node dysfunction with bradycardia. She had episodes of sinus arrest with idioventricular escape rhythm. Since she had persistent left ventricular dysfunction with an ejection fraction of 25%, an implantable cardioverter-defibrillator (ICD) was done. Nine months later she had erosion of the ICD pulse generator in the absence of infection and needed explanation followed by reimplantation on the contralateral side. Six years after the initial presentation she was diagnosed with symptomatic typical cavo-tricuspid isthmus dependent atrial flutter that required ablation. Seven years after the initial presentation, she developed left bundle branch block and underwent upgrade of the ICD to a biventricular cardioverter defibrillator. The patient stated that her deceased mother, a son, and her grandson may have the disease. We offered genetic studies for the son and grandson but both declined to have testing.

## Discussion

Apart from HIV-related lipodystrophy, the other lipodystrophic syndromes are rare, from some dozens to some hundreds of cases reported, depending of the subtype.^1,3^ Lipodystrophies can be congenital or acquired, and generalized or partial. Type 2 FPLD is a Mendelian disease characterized by loss of subcutaneous adipose tissue from the extremities and trunk and its deposition in the head and neck. In women, the phenotype usually arises during puberty. Insulin resistance, diabetes, hypercholesterolemia, hypertriglyceridemia, and low high-density lipoprotein can be associated with this disease as has been in our case. The extent of loss of adipose tissue correlates with the severity of associated metabolic complications. Metabolic complications, insulin resistance, diabetes, and dyslipidemia are often more prominent in women. Atherosclerotic disease including coronary artery disease is also more prominent among women. Atherosclerotic disease is thought to be due to the presence of risk factors rather than due to a direct effect of the disease. The Dunnigan variety, or FPLD type 2, is the most common type of FPLD and is generally due to missense mutations in *LMNA* gene located on chromosome 1q21-22.^4^ Normal adipose tissue distribution at birth and redistribution in puberty is the characteristics of this disorder. Although these patients have prominent muscular build, this disease does not result in gain of muscle strength.

Mutations in *LMNA* are much commonly reported to be associated with muscular dystrophy and/or cardiomyopathy than FPLD2. Furthermore, mutations in *LMNA* resulting in FPLD2 do not commonly result in cardiomyopathy. Concurrence of both cardiomyopathy and/or conduction defects and FPLD2 due to mutations in *LMNA* is very rare, but some cases have been reported.^3,5^

Seventy-five percent patients with FPLD2 have mutation in a single allele encoding the arginine residue at position 482, as in our case. These patients develop the classic components of FPLD: partial lipodystrophy, insulin resistance, and other complications. However, cardiomyopathy is rare. Although *LMNA* mutations are associated with both cardiomyopathy and FPLD, most patients with FPLD do not develop cardiomyopathy. Interestingly, most mutations in patients with FPLD occur in the carboxy terminal domain of lamin A/C, while cardiomyopathy has been associated with mutations in amino terminal domain of the lamin A/C in FPLD patients.^2^ Several heterozygous mutations in *LMNA* like the R28W, R60G, R62G, and D192V have been reported to be associated with lipodystrophy and cardiomyopathy. Some mutations have been associated with conduction system abnormalities as well.^2^ A strong association has been reported between mutations in *LMNA* gene exons 1 and 3 with typical FPLD and cardiomyopathy, especially dilated cardiomyopathy.^2^ Mutations in *LMNA* gene exon 9 have been associated with FPLD, cardiac arrhythmias and muscular dystrophy,^6^ and mutations in *LMNA* gene exon 11 have been associated with FPLD, hypertrophic cardiomyopathy, and aortic stenosis.^5^ Patients with mutations in exons 1 and 3 have been reported to have similar pattern of body fat loss as observed in patients with typical FPLD with mutations in exon 8, as well as the other phenotypical manifestations such as insulin resistance, diabetes, hepatic steatosis, hypertriglyceridemia, acanthosis nigricans, and polycystic ovarian syndrome.^2^

Apart from FPLD2, mutations in *LMNA* gene has been reported to be associated with a wide variety of other disorders including childhood-onset generalized lipodystrophy, muscular dystrophies, neuropathy, hand heart syndrome, mandibuloacral dysplasia, and Hutchinson-Gilford Progeria syndrome. Similarly mutations in almost all the exons of *LMNA* gene have been known to be associated with cardiomyopathy without FPLD.^7^ *LMNA* mutation is the most common gene defect associated with dilated cardiomyopathy identified in about 8% of patients and is associated with a poor prognosis. The association with *LMNA* mutations is much more common in patients with dilated cardiomyopathy and conduction system disorders being present in 30% of such subsets.

There are some *LMNA* mutations that are associated with early onset and severe cardiac disease including conduction system disease needing pacemaker implants and cardiomyopathy requiring transplantations. As stated above, dilated cardiomyopathy with mutations in *LMNA* are associated with poor prognosis. On a medial follow-up of 57 months on 60 affected members, there were 15 heart transplants, 15 sudden cardiac arrests, 12 defibrillator implants, and 1 death from end-stage cardiac failure.^8^ Data from ICD implantations done in patients with *LMNA* mutations needing pacemaker implants (with mean ejection fraction 58%) showed that 42% of these subjects received appropriate therapy for ventricular tachycardia and ventricular fibrillation, indicating that these patients are at high risk for developing arrhythmias. The authors conclude that ICD implantation rather than pacemaker should be considered for primary prevention in patients with *LMNA* mutations needing pacemaker implantation. At present, a pathophysiological link between FPLD2 with primary hyperparathyroidism and euthyroid multinodular goiter, all present in our patient, is unknown.

## Conclusion

In summary, this patient presented with several classic features of familial partial lipodystrophy and cardiac complications described in those patients including nonischemic cardiomyopathy, aggressive coronary artery disease, conduction system disease requiring pacemaker implant, and atrial flutter. FPLD is important for the internists and cardiologists to consider in differential diagnosis when apparently muscular looking patients present with dilated cardiomyopathy, insulin resistance, diabetes mellitus, and dyslipidemia. The characteristic phenotype helps in recognition of this disease, but physicians need to be aware of this to make the diagnosis. One life-threatening complication of this condition is cardiomyopathy, the risk of which is greater in women.
